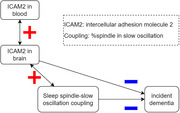# The Role of Blood‐Based Proteomic Biomarkers in Sleep Spindle‐Slow Oscillation Coupling and Incident Dementia

**DOI:** 10.1002/alz.089218

**Published:** 2025-01-09

**Authors:** Zachary Xin Chen, Can Zhang, Prasenjit Mondal, Bruno A. Benitez, Haoqi Sun

**Affiliations:** ^1^ Boston Latin School, Boston, MA USA; ^2^ Massachusetts General Hospital, Charlestown, MA USA; ^3^ Beth Israel Deaconess Medical Center, Boston, MA USA; ^4^ Massachusetts General Hospital, Boston, MA USA

## Abstract

**Background:**

Sleep is impaired in dementia. Therefore, sleep has been proposed as an intervention target for incident dementia. The microstructures of sleep electroencephalography (EEG), such as spindle, slow oscillation (SO), and their coupling, have neuronal bases and functions in learning and memory. Higher spindle‐SO coupling is linked to lower risk of incident dementia in epidemiological studies. Here, we aim to use proteomics to explore the molecular basis of this link.

**Method:**

This retrospective study used the Framingham Heart Study (FHS) Offspring and OMNI 1 Cohorts, accessible through BioLINCC. The inclusion criteria are (1) plasma proteomics available, where the blood was drawn at FHS‐Exam5 and profiled using SOMAscan (1373 proteins); (2) sleep EEGs from the Sleep Heart Health Study on National Sleep Research Resources obtained at FHS‐Exam6. The exclusion criteria are (1) missing covariates: age, sex, education, APOE ε4 allele, body mass index, and smoking; (2) pre‐existing dementia before sleep recording. Spindle‐SO coupling was defined as the fraction of spindles within SO at N2 or N3 stages of sleep, detected using Luna. Incident dementia was based on FHS consensus. We performed a proteome‐wide association study (PWAS) for spindle‐SO coupling using Spearman’s correlation. We also performed PWAS for incident dementia using Fine‐Gray survival analysis with death as competing risk. We then found the common proteins between the two PWASes. Since the proteins were from the blood, we filtered them by assessing their blood‐brain expression correlations using Genotype‐Tissue Expression.

**Result:**

The PWAS for incident dementia had n=1048 (52% female) with maximum 47‐year follow‐up, revealing 35 proteins reaching Bonferroni‐corrected significance. In contrast, the PWAS for spindle‐SO coupling had n=146 (57% female), with no Bonferroni‐corrected significance. There were 28 proteins common to both PWASes before Bonferroni correction. Among them, intercellular adhesion molecule 2 (ICAM2), C‐reactive protein, and myoglobin demonstrated significant blood‐brain correlations, with ICAM2 exhibiting brain‐wide expression (Figure 1). One standard deviation increase in ICAM2 is associated with hazard ratio of 0.48 (95% confidence interval 0.23‐0.99, p=0.048) for incident dementia. ICAM2 and spindle‐SO coupling have Spearman’s correlation at 0.24 (0.08‐0.39, p=0.0036).

**Conclusion:**

ICAM2 may serve as the proteomic basis for the association between spindle‐SO coupling and incident dementia.